# Reconstruction Versus Hemiarthroplasty in Comminuted (Three- and Four-Part) Proximal Humerus Fractures: A Retrospective Functional Outcome Analysis at 6 Months

**DOI:** 10.3390/clinpract16020030

**Published:** 2026-01-29

**Authors:** Alexandru Lisias Dimitriu, Monica Georgiana Roman, Elisa Georgiana Popescu, Eduard Cătălin Georgescu, Dragoș Ene, Răzvan Ene

**Affiliations:** 1 Department 14 Orthopedics-Intensive Care, “Carol Davila” University of Medicine and Pharmacy, 050474 Bucharest, Romania; alexandru.dimitriu@umfcd.ro (A.L.D.); elisa-georgiana.popescu@drd.umfcd.ro (E.G.P.); eduard-catalin.georgescu@drd.umfcd.ro (E.C.G.); dragos.ene@umfcd.ro (D.E.); razvan.ene@umfcd.ro (R.E.); 2 Department of Orthopaedics, Clinical Emergency Hospital, 014461 Bucharest, Romania; 3 Department of Surgery, Clinical Emergency Hospital, 014461 Bucharest, Romania

**Keywords:** proximal humerus fractures, open reduction and internal fixation, shoulder hemiarthroplasty, tuberosity healing, functional outcome assessment, elderly patients

## Abstract

**Background:** The optimal management of comminuted proximal humerus fractures in the elderly remains controversial. Although hemiarthroplasty is widely used for complex fracture patterns, its functional superiority over reconstruction is not consistently demonstrated. The aim of this study was to compare early functional outcomes following open reduction and internal fixation (ORIF) versus hemiarthroplasty in elderly patients with three- and four-part proximal humerus fractures. **Methods:** This retrospective single-center study included elderly patients with comminuted proximal humerus fractures treated between 2020 and 2024 by either ORIF or hemiarthroplasty. Functional outcomes were assessed at 6 months using the Constant–Murley and DASH scores. Secondary outcomes included complication rates, range of motion, and early reintervention. **Results:** At 6 months, the ORIF group showed a mean Constant–Murley score of 62.1 ± 9.4 compared with 58.0 ± 10.2 in the hemiarthroplasty group. DASH scores were 34.2 ± 10.8 for ORIF and 38.5 ± 11.3 for hemiarthroplasty. Pain levels were similarly low in both groups (VAS 2.6 ± 1.1 vs. 2.9 ± 1.2). Complication rates were comparable, with fixation-related issues occurring in 17% of ORIF cases and tuberosity-related complications in 11% of hemiarthroplasty cases. **Conclusions:** Hemiarthroplasty should not be regarded as the default treatment strategy for comminuted proximal humerus fractures in elderly patients. When stable anatomic reduction is achievable, ORIF can yield comparable early functional results, emphasizing that patient selection and tuberosity management remain more important than the choice of implant.

## 1. Introduction

Comminuted fractures of the proximal humerus, particularly three- and four-part injuries as described by Neer, remain among the most challenging upper-limb fractures to manage in elderly patients [1]. The increasing incidence in the aging population, combined with osteoporotic bone and frequent medical comorbidities, has led to a progressive shift in many centers toward arthroplasty-based solutions [2]. Hemiarthroplasty has traditionally been considered an acceptable option for pain control and early mobilization in severe fracture patterns, particularly when anatomical reconstruction is difficult or tuberosity healing is uncertain [3].

However, recent studies have shown that functional outcomes after hemiarthroplasty depend heavily on tuberosity healing, implant positioning, and postoperative rehabilitation—factors that are not always more predictable than with reconstruction. At the same time, advances in angular-stable locking plates, improved intraoperative imaging, and better soft-tissue handling techniques have allowed reconstruction (ORIF) to remain a relevant treatment alternative, even in elderly patients, when fracture fragments are reconstructible and bone stock is adequate [4]. In selected cases, particularly when the tuberosities can be anatomically restored, ORIF may offer functional outcomes that are comparable to or even superior to hemiarthroplasty [5].

The current literature presents variability in real-world outcomes, and it remains unclear whether hemiarthroplasty should be considered the default solution for all comminuted fractures in elderly patients [6]. Moreover, most comparative studies focus on pain or radiographic outcomes, while fewer provide detailed functional assessment using more than one validated scoring system [7]. There is also limited evidence from Level I trauma centers in Eastern Europe, where reverse arthroplasty is not yet universally available, and reconstructive strategies continue to play a central role in clinical decision-making.

Therefore, the aim of this retrospective study was to compare short-term functional outcomes of ORIF versus hemiarthroplasty in elderly patients with three- and four-part proximal humerus fractures, treated at a Level I trauma center in Bucharest, Romania, between 2020 and 2024. Functional evaluation was performed using both the Constant–Murley and DASH scores at 6 months. We hypothesized that when anatomical reconstruction is achieved and tuberosities are preserved, ORIF can still yield competitive functional outcomes compared to hemiarthroplasty, despite the increasing global trend toward primary arthroplasty in this fracture setting.

## 2. Materials and Methods

### 2.1. Study Design

This study was designed as a retrospective, observational, single-center analysis comparing outcomes of reconstruction (ORIF) versus hemiarthroplasty in elderly patients with comminuted proximal humerus fractures. All eligible patients treated between 2020 and 2024 at a Level I trauma center in Bucharest, Romania, were screened for inclusion.

### 2.2. Patient Selection

Inclusion criteria were: age ≥ 60 years; displaced three- or four-part proximal humerus fractures classified according to the Neer system. AO/OTA 2018 codes were used to characterize fracture complexity (most corresponding to 11-B or 11-C patterns), but the Neer classification served as the primary system for patient selection, given that AO/OTA type C fractures frequently—but not always—align with Neer four-part configurations.

Definitive surgical treatment with ORIF or hemiarthroplasty; and availability of functional assessment at 6 months (±30 days). Exclusion criteria were: pathological fractures, associated major ipsilateral injuries preventing assessment, previous neurologic impairment of the affected limb, revision surgery before 6 months, loss to follow-up, or incomplete medical records. A total of 40 patients met the eligibility criteria (18 ORIF and 22 hemiarthroplasty). All patients included in the study were older adults (62–89 years), and no younger individuals met the eligibility criteria during the study period.

### 2.3. Surgical Decision-Making

In our Level I trauma center, treatment allocation followed standard institutional practice. ORIF was attempted whenever anatomical reduction was considered feasible based on preoperative radiographs and CT imaging, with adequate metaphyseal bone stock and reconstructible tuberosities. Hemiarthroplasty was chosen in cases with marked tuberosity comminution, severe osteoporotic bone, head-splitting patterns, or when stable fixation was deemed unlikely. The final decision was made intraoperatively by the attending senior trauma surgeon after assessing reducibility and fixation stability under direct visualization and fluoroscopy. This pathway reflects real-world practice and explains the non-randomized nature of treatment allocation.

### 2.4. Surgical Technique

All procedures were performed through a standard deltopectoral approach.

In the ORIF group, anatomical reduction was attempted whenever feasible, followed by fixation using a precontoured proximal humerus locking plate (Double Medical, Zhangzhou, China, 3.5 mm angular-stable system). Tuberosities were secured with No. 2 nonabsorbable sutures passed through the plate or via bone tunnels. Structural allograft (cancellous allograft chips) was used selectively in cases presenting with metaphyseal bone loss, medial column deficiency, or insufficient calcar support where additional biological augmentation was necessary to maintain reduction. No autograft or synthetic bone substitutes were used in this cohort.

In the hemiarthroplasty group, all patients received an uncemented, press-fit modular humeral stem (Zimmer Biomet, Warsaw, IN, USA). In every case, humeral height, offset, and retroversion were restored according to manufacturer guidelines. The greater and lesser tuberosities were repaired using heavy nonabsorbable sutures in a standardized four-suture configuration to optimize healing. Intraoperative fluoroscopy was used in both groups.

### 2.5. Postoperative Care and Rehabilitation

All patients received standard perioperative antibiotic prophylaxis and thromboprophylaxis according to institutional guidelines [8]. Postoperative rehabilitation followed a unified institutional protocol applied identically to both treatment groups: shoulder immobilization in a sling for 2–4 weeks depending on intraoperative stability, followed by early passive-assisted mobilization, progression to active motion at 4–6 weeks, and strengthening after 8–10 weeks. The rehabilitation pathway was supervised by physiotherapists experienced in upper limb trauma recovery. Due to the retrospective design of the study, individual adherence to the rehabilitation protocol (session attendance or home-exercise compliance) could not be reliably quantified.

### 2.6. Assessment of Tuberosity Healing

Tuberosity healing was assessed using routine postoperative radiographs obtained at follow-up visits (anteroposterior and scapular Y views). Healing was defined as trabecular continuity between the tuberosities and the humeral metaphysis, absence of secondary displacement compared with immediate postoperative imaging, and maintenance of implant stability. Malunion or nonunion was recorded when persistent fragmentation, resorption, or displacement greater than 5 mm was identified.

Clinical assessment supplemented radiographic evaluation and included localized tenderness, external rotation strength, and range of motion. Computed tomography was not routinely performed and was reserved for cases in which clinical or radiographic findings raised suspicion of nonunion.

### 2.7. Outcomes and Data Collection

The primary outcomes were functional results at 6 months, assessed using the Constant–Murley score [9] and the Disabilities of the Arm, Shoulder and Hand (DASH) score [10]. Secondary outcomes included pain intensity (VAS 0–10) [11], range of motion (forward flexion, abduction, external rotation), complications (infection, tuberosity nonunion/malunion, head necrosis, stiffness), and reintervention or revision surgery. Demographic and perioperative variables (age, sex, fracture pattern, operative time, and length of hospital stay) were also recorded.

### 2.8. Statistical Analysis

Normality of distribution was evaluated using the Shapiro–Wilk test. Continuous variables were compared using an independent-samples *t* test or Mann–Whitney U test, as appropriate. Categorical variables were compared using the χ^2^ test or Fisher’s exact test. A clinically relevant difference was predefined as ≥10 points for both Constant and DASH scores. Effect sizes with 95% confidence intervals were reported when applicable. Statistical significance was set at *p* < 0.05. All analyses were performed using IBM SPSS Statistics, Version 26.0 (IBM Corp., Armonk, NY, USA).

### 2.9. Ethics

The study was conducted in accordance with the Declaration of Helsinki and approved by the Institutional Ethics Committee of the Clinical Emergency Hospital Bucharest. The Committee reviewed the study protocol and confirmed that it met the criteria for minimal-risk retrospective research based exclusively on fully anonymized clinical data. Accordingly, the Committee granted a formal waiver of individual informed consent. All patient information was anonymized prior to analysis. A written approval document is available and can be provided to the Editorial Office upon request.

### 2.10. Use of Artificial Intelligence Tools

During the preparation of this manuscript, the authors used ChatGPT (GPT-5, OpenAI, 2025) exclusively for language refinement and editorial support. The AI tool was not used for data generation, data analysis, statistical processing, or interpretation of results. All content was critically reviewed and approved by the authors, who take full responsibility for the accuracy and integrity of the manuscript.

## 3. Results

A total of 40 patients were included, of whom 18 underwent reconstruction with ORIF and 22 underwent hemiarthroplasty. Baseline characteristics were comparable between groups regarding age, sex distribution, fracture severity (three- vs. four-part), time to surgery, and length of hospital stay (Table 1). Operative time was significantly longer in the ORIF group compared with the hemiarthroplasty group (92 ± 18 min vs. 80 ± 15 min, *p* = 0.014).

At 6 months, patients treated with ORIF demonstrated slightly higher Constant–Murley scores compared with the hemiarthroplasty group (62.1 ± 9.4 vs. 58.0 ± 10.2), corresponding to a small mean difference of approximately 4 points, which did not reach statistical significance. Similarly, DASH scores were marginally lower (better) in the ORIF group (34.2 ± 10.8 vs. 38.5 ± 11.3), again without statistical significance (Table 2). Pain levels were low in both cohorts (VAS ≈ 2–3), and range of motion favored ORIF by a small margin in forward flexion and abduction.

**Figure 1:**
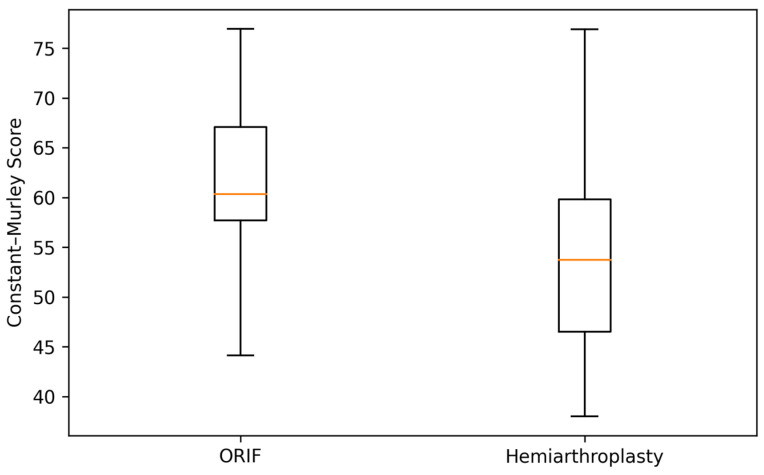
Distribution of Constant–Murley scores at 6 months. Boxplot illustrating the distribution of Constant–Murley scores at 6 months in the ORIF and hemiarthroplasty groups. The marked overlap demonstrates the absence of a clinically meaningful difference in early functional outcome.

**Figure 2:**
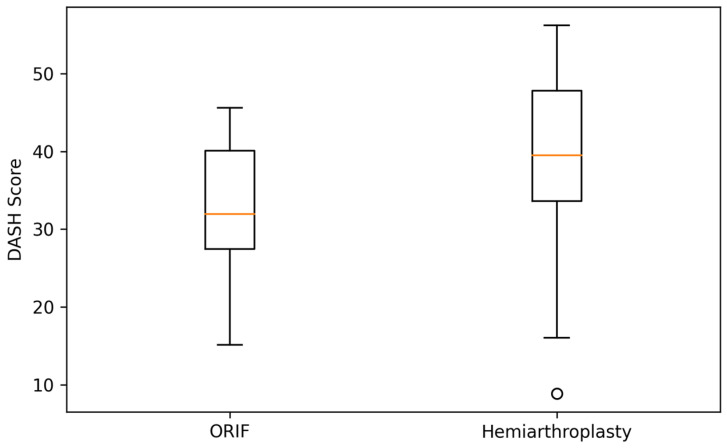
Distribution of DASH scores at 6 months. Boxplot showing the distribution of DASH scores at 6 months. Although median values favored ORIF, substantial overlap between groups indicates comparable early recovery.

To complement the tabulated results, Figure 1 and Figure 2 illustrate the distribution of Constant–Murley and DASH scores at 6 months. Both boxplots demonstrate substantial overlap between the ORIF and hemiarthroplasty groups, visually reinforcing the absence of statistically significant or clinically meaningful differences in early functional outcomes.

Complication rates were overall low in both treatment arms. ORIF was associated with a slightly higher proportion of fixation-related technical complications (loss of reduction/implant-related issues: 17% vs. 8%), whereas hemiarthroplasty showed a modestly higher rate of tuberosity-related problems (11% vs. 6%). Rates of stiffness, infection, humeral head necrosis, and reintervention were similar between groups, with no statistically significant differences (Table 3). No periprosthetic fractures or deep infections leading to implant removal were observed in this cohort.

## 4. Discussion

Management of complex three- and four-part proximal humerus fractures in elderly patients remains controversial, despite the increasing global trend toward arthroplasty-based treatment. Hemiarthroplasty has historically been promoted as a reliable solution for pain control and early mobilization when reconstruction is difficult, particularly in patients with osteoporotic bone or comminution of the tuberosities. However, recent comparative studies suggest that functional recovery after hemiarthroplasty is highly dependent on tuberosity healing and restoration of shoulder biomechanics, and not merely on the implant itself. Consequently, the assumption that arthroplasty is inherently superior to reconstruction has been increasingly questioned in the literature, especially when modern fixation techniques and proper case selection make anatomic reduction achievable.

In the present cohort, ORIF demonstrated slightly better functional outcomes than hemiarthroplasty at 6 months, although these differences were small and did not reach statistical significance. These findings suggest that, in carefully selected cases where anatomical reconstruction is feasible, ORIF can achieve early functional results that approximate those of hemiarthroplasty, rather than indicating any proven superiority. Pain levels and early range of motion were also comparable between groups, supporting the interpretation that short-term recovery may be influenced more by fracture biology and rehabilitation than by implant choice alone.

The present findings also reinforce the idea that hemiarthroplasty does not inherently guarantee superior functional recovery in elderly patients with complex proximal humerus fractures [12]. Although it is frequently perceived as a more “definitive” solution for comminuted patterns, its success is largely conditioned by tuberosity healing, implant positioning, and soft-tissue balance [13]. In clinical practice, these variables are not always more predictable than with reconstruction, particularly when bone quality permits stable fixation [14]. As a result, hemiarthroplasty should not be viewed as a default strategy based solely on fracture complexity, but rather as an option reserved for cases in which anatomical reconstruction is not achievable [15].

A key element explaining the comparable outcomes between the two strategies is the central role of tuberosity healing in postoperative shoulder function. In hemiarthroplasty, poor tuberosity integration can neutralize the theoretical biomechanical advantage of the prosthesis and result in restricted motion, impaired external rotation, and lower functional scores [16]. Conversely, when stable fixation permits anatomic restoration of the proximal humerus and the rotator cuff insertions, reconstruction can preserve native biomechanics and allow a more physiological recovery trajectory [17]. From a functional standpoint, therefore, the quality of tuberosity management may outweigh the theoretical benefit of replacing the humeral head, particularly in the early postoperative period [15].

The treatment algorithm applied in this study reflects the real-world constraints and decision-making of a Level I trauma center in which reverse shoulder arthroplasty is not routinely used for acute fractures. In this setting, hemiarthroplasty is reserved for cases where bone quality or fragment comminution prevents stable reconstruction, whereas ORIF remains the preferred option whenever an anatomical reduction is technically achievable. This pragmatic approach highlights that the choice between preservation and replacement is often driven not by the implant itself, but by the feasibility of restoring native anatomy. Under these conditions, the functional parity observed between the two strategies supports the concept that adequate case selection remains more important than implant escalation.

The choice of a six-month follow-up reflects the standardized postoperative pathway in our institution, where all patients undergo formal functional scoring at this interval. This timepoint provides a consistent early indicator of recovery, but does not allow conclusions regarding medium- or long-term divergence between treatment strategies. As such, the findings of this study should be interpreted as representative of early postoperative outcomes, and not extrapolated to longer-term shoulder function or implant longevity.

The predefined threshold of a 10-point difference in Constant–Murley and DASH scores was based on previously published estimates of the minimal clinically important difference (MCID) for shoulder function, which generally range from 8–10 points for the Constant–Murley score and 10–13 points for the DASH score in older adults following traumatic or reconstructive shoulder procedures. Using a 10-point cutoff therefore represents a conservative and clinically meaningful benchmark for interpreting early functional differences.

We also acknowledge that the study’s modest sample size (*n* = 40) limits statistical power, particularly for detecting smaller effect sizes. As a result, the findings should be interpreted as exploratory and reflective of real-world trends rather than definitive comparative estimates. Larger prospective cohorts would be required to determine with greater precision whether subtler differences exist between treatment modalities.

This study has several limitations that should be acknowledged. First, its retrospective design introduces an inherent degree of selection bias, as the choice between ORIF and hemiarthroplasty was based on intraoperative feasibility rather than randomization. Second, the follow-up period was limited to 6 months, capturing early functional recovery but not potential medium- or long-term divergence as tuberosity remodeling, rotator cuff function, and implant-related changes evolve over time. Third, a further limitation is the absence of reverse shoulder arthroplasty (RSA) in the treatment algorithm. Although RSA has become increasingly prominent in global practice for complex proximal humerus fractures, it was not routinely available in our center during the study period. This restricts the generalizability of our findings to settings where RSA is not consistently accessible, and outcomes may differ in healthcare systems where RSA is the predominant treatment pathway. Nevertheless, the present results offer meaningful insight into real-world surgical decision-making in elderly patients with comminuted fractures, illustrating that hemiarthroplasty is not inherently superior when anatomical reconstruction is technically achievable. Future prospective studies with longer follow-up and inclusion of reverse arthroplasty would help clarify the durability of these early functional trends and further refine patient selection criteria.

Although the findings may reflect real-world surgical decision-making in a Level I trauma center, they should be interpreted with caution given the retrospective design, selection bias, the absence of RSA as a comparator, and the limited sample size. The trends observed here may help guide expectations, but they do not establish equivalence between ORIF and hemiarthroplasty, nor do they define optimal treatment thresholds.

## 5. Conclusions

Hemiarthroplasty should not be assumed to provide superior early functional outcomes for all elderly patients with comminuted proximal humerus fractures. In cases where anatomical reconstruction is achievable and tuberosity fixation is stable, ORIF can offer comparable short-term functional recovery.

However, given the retrospective design, limited sample size, and absence of RSA in the treatment algorithm, these findings should be interpreted as exploratory. Treatment decisions should remain individualized, based on fracture morphology, bone quality, and surgeon expertise. Larger prospective studies are needed to more definitively compare the functional trajectories of ORIF and hemiarthroplasty in this population.

## Figures and Tables

**Table 1:** Baseline characteristics of the study population. This table summarizes demographic and perioperative characteristics of patients treated with ORIF or hemiarthroplasty.

Variable	ORIF (*n* = 18)	Hemiarthroplasty (*n* = 22)	*p*-Value
Age (years), mean ± SD	73.1 ± 7.0	75.2 ± 7.1	0.270
Female, *n* (%)	12 (67%)	15 (68%)	0.946
4-part fractures, *n* (%)	12 (67%)	15 (68%)	0.946
AO/OTA C1, *n* (%)	10 (56%)	12 (55%)	0.94
AO/OTA C2, *n* (%)	8 (44%)	8 (36%)	0.56
AO/OTA C3, *n* (%)	0 (0%)	2 (9%)	0.18
Time to surgery (days)	3.2 ± 1.2	3.4 ± 1.5	0.610
Operative time (min)	92 ± 18	80 ± 15	0.014
Hospital stay (days)	5.1 ± 1.8	5.4 ± 2.0	0.571

Values are expressed as mean ± standard deviation for continuous variables and as number (percentage) for categorical variables.

**Table 2:** Functional outcomes at 6 months. This table compares postoperative functional outcomes between groups at 6 months following surgery.

Outcome	ORIF (*n* = 18)	Hemiarthroplasty (*n* = 22)	*p*-Value
Constant–Murley score	62.1 ± 9.4 (95% CI 57.4–66.8)	58.0 ± 10.2 (95% CI 53.6–62.4)	0.180
DASH score	34.2 ± 10.8 (95% CI 29.0–39.4)	38.5 ± 11.3 (95% CI 33.6–43.4)	0.230
VAS pain	2.6 ± 1.1 (95% CI 2.1–3.1)	2.9 ± 1.2 (95% CI 2.4–3.4)	0.300
Forward flexion (°)	125 ± 20 (95% CI 115–136)	118 ± 22 (95% CI 108–128)	0.220
Abduction (°)	115 ± 22 (95% CI 104–126)	108 ± 24 (95% CI 97–119)	0.250
External rotation (°)	38 ± 12 (95% CI 32–44)	34 ± 12 (95% CI 29–39)	0.270

Values are expressed as mean ± standard deviation. Positive values for the mean difference represent better outcomes in the ORIF group; negative values favor hemiarthroplasty.

**Table 3:** Complications in the two treatment groups. This table reports the distribution of early complications observed in each treatment group.

Complication	ORIF (*n* = 18)	Hemiarthroplasty (*n* = 22)	*p*-Value
Superficial infection	1 (6%)	1 (5%)	0.880
Deep infection	0 (0%)	0 (0%)	1.000
Loss of reduction/implant-related	3 (17%)	2 (8%)	0.390
Tuberosity nonunion/malunion	1 (6%)	2 (11%)	0.620
Stiff shoulder	2 (11%)	3 (14%)	0.790
Reintervention/revision	2 (11%)	1 (5%)	0.520

Values are expressed as number (percentage). No statistically significant differences were observed between groups.

## Data Availability

The data presented in this study are available on request from the corresponding author due to ethical and privacy restrictions.
